# Dietary Soybean Oil Supplementation Affects Keel Bone Characters and Daily Feed Intake but Not Egg Production and Quality in Laying Hens Housed in Furnished Cages

**DOI:** 10.3389/fvets.2021.657585

**Published:** 2021-03-19

**Authors:** Haidong Wei, Lei Pan, Chun Li, Peng Zhao, Jianhong Li, Runxiang Zhang, Jun Bao

**Affiliations:** ^1^College of Animal Science and Technology, Northeast Agricultural University, Harbin, China; ^2^College of Life Science, Northeast Agricultural University, Harbin, China; ^3^Key Laboratory of Chicken Genetics and Breeding, Ministry of Agriculture and Rural Affairs, Harbin, China

**Keywords:** laying hen, keel bone health, production performance, soybean oil, diet

## Abstract

To evaluate dietary soybean oil supplementation on production performance, egg quality, and keel bone health in laying hens. Two hundred and four laying hens at 20 weeks of age (WOA) were distributed into 12 cages containing 17 birds each. Birds were either fed a commercial diet (control group, CON) or a diet supplemented with 3% of soybean oil (SO group). Experiments lasted 17 weeks. Body weight, daily feed intake, production performance and egg quality were measured at 25, 29, 33, and 37 WOA. Birds were subsequently assessed for keel bone status by palpation, and keel was excised to measure bone length, microstructure, bone mineral density (BMD), elements contents, and the expression of osteoprotegerin (OPG), receptor activator of nuclear factor kappa-B ligand (RANKL), collagen type II alpha 1 (COL2α1), periostin (POSTN), and sclerostin (SOST). The results showed that dietary SO supplementation did not affect production performance and egg quality (*P* > 0.05), but improved body weight of hens at 29 and 37 WOA (*P* < 0.05), and decreased daily feed intake at 33 and 37 WOA (*P* < 0.05). Incidence of keel bone damage (especially fracture) was higher in hens of SO group. Keel bone length in birds of SO group was significantly decreased compared to CON (*P* < 0.05). Keel bone of supplemented hens showed increased trabecular separation at 29 WOA and higher levels of V, Mn, Fe, Se, and Ba at 33 WOA (*P* < 0.05). Moreover, decreased BMD, trabecular number and thickness were observed in keel bone of laying hens receiving supplementation at 29 and 37 WOA (*P* < 0.05); decreased levels of Li, Ca, Hg, and TI at 33 WOA and trabecular thickness at 37 WOA (*P* < 0.05) were also identified. mRNA levels of SOST and RANKL and the ratio of RANKL/OPG mRNA levels were increased in birds fed a SO-supplemented diet (*P* < 0.05); COL2α1, OPG, and POSTN were downregulated at all sampling points (*P* < 0.05). Taken together, results indicate that feeding laying hens a diet supplemented with soybean oil can decrease daily feed intake and impair keel bone health but not influence production performance and egg quality.

## Introduction

The incorporation of fats and oils has become routine in modern poultry production as a way to increase energy value of the diet. Oil-enriched diets have greater palatability, reduced dust content, and lowered passage rate through the intestinal tract in poultry, allowing more time for nutrients to be fully absorbed (1). As other oils of vegetable origin, soybean oil (SO) contains a large amount of essential fatty acids, and it is commonly added to poultry diets. Previous studies have shown that SO supplementation in laying hen diets led to improved egg quality as revealed by increased daily egg weight and mass production (2, 3). However, other studies have indicated that providing a SO-supplemented diet to laying hens did not significantly improve production performance or egg quality (4, 5). Broilers fed with SO-enriched diets showed increased body weight without major impact on bone quality (6). Interestingly, Jiang et al. have described reduced bone weight, strength, and serum calcium content in laying hens fed with SO-supplemented diets (7), suggesting that incorporation of SO into diets can impact bone quality. Therefore, layering hen diets containing high contents of SO can have adverse effects on eggshell quality and bone health.

The keel bone is a prolongation of the sternum that runs axially accompanying the midline, and plays a key role in aiding flight and breathing in birds. Various external factors can affect keel bone integrity, such as housing systems, nutrition levels, and available space for movement (8). Furthermore, some studies found that laying hens with high laying performance had more severe keel bone fractures (9), thus egg production also negatively affects keel bone heath. Keel bone damage (KBD), including fractures and deviations, is a health and welfare concern in laying hen farms. Previous studies have reported that keel bone fractures in hens led to altered behavior, reduced motion, enhanced stress response, and decreased egg production, egg weight and shell quality (10–12). Therefore, KBD has a detrimental impact on welfare and production performance in laying hens.

X-ray microtomography (Micro-CT) has been widely used to evaluate microarchitecture of trabecular bones in animals (13). Evaluated bone microstructure parameters include bone volume fraction (BV/TV), trabecular thickness (Tb.Th), trabecular separation (Tb.Sp), trabecular number (Tb.N), and connectivity density (Conn.Dn). Determination of bone microstructure and bone mineral density (BMD) provides an overall understanding of bone health state and stiffness (14). Results from our previous studies revealed that keel bone fractures in laying hens determine alteration in bone quality leading to decreased BV/TV, Tb.N, degree of anisotropy (DA), and Conn.Dn, and increased Tb.Sp (15). Aguado et al. reported that hypo-activity also leads to decreased femur quality and altered trabecular microarchitecture in chickens, as revealed by decreased Tb.N and increased Tb.Sp (16). Furthermore, altered bone quality is accompanied by mineral elements homeostatic imbalance in laying hens, especially calcium (Ca), phosphorus (P), copper (Cu), and boron (B) (15).

The receptor activator of nuclear factor-κB ligand (RANKL), also known as osteoclast differentiation factor, is produced by osteoblasts and plays a vital role in osteoclastogenesis by stimulating osteoclast activation, formation and differentiation (16). Osteoprotegerin (OPG), or osteoclastogenesis inhibitory factor, is produced by different cell types other than osteoblasts and hinders differentiation of osteoclast precursors into mature osteoclasts, acting as a natural decoy receptor for RANKL, hence balancing RANKL-induced osteoclastic activity and attenuating bone resorption (17). Collagen type II alpha 1 (COL2α1) is an important collagen-specific protein produced in chondroblasts that is essential to the regulation of bone turnover in laying hens (18). Periostin (POSTN) and sclerostin (SOST) are also involved in establishing bone quality and pathophysiological changes in laying hens on a low calcium diet (19). Collectively, these molecules have a unique function in maintaining bone health and quality.

To the best of our knowledge, it is still poorly understood the influence of dietary soybean oil supplementation on keel bone characters in laying hens housed in furnished cages. Hence, the aim of this study was to examine the effects of soybean oil added to the diet of laying hens on growth and production performance, egg quality, and keel bone characters consist of the rate of KBD, bone metabolism and remodeling, bone mineral elements homeostasis, microstructure, and mineral density. We hypothesized that dietary SO supplementation can improve egg production and egg quality, and affect daily feed intake and keel bone characters in laying hens housed in furnished cages.

## Materials and Methods

### Birds and Experimental Design

Two hundred and four healthy Lohmann white laying hens presenting normal keel bones at 20 weeks of age (WOA) weighing 1.09 ± 0.05 kg were included in this study. Birds were randomly divided into two groups: control (CON) group and SO-supplemented (SO) group, each composed by six cages containing 17 birds. Laying hens in CON group were fed a commercial basal diet, whereas a basal diet supplemented with 3% SO was given to animals in SO group. Ingredients and nutritional content of experimental diets are listed in Table 1. Laying hens in each replicate were housed in similar furnished cages. Cages dimensions were 180 cm × 70 cm × 70 cm (length × width × height); cages design consisted of a wooden square perch, an elevated nest box, five nipple drinkers, and a rectangular feeder. All birds had free access to feed and water throughout the duration of the experiment, i.e., from 20 to 37 WOA. Housing temperature was 20–25°C, and relative humidity was 45–60%.

**Table 1:** Ingredients and nutrient composition of the diets.

**Items**	**CON group**	**SO group**
**Ingredient (%)**		
Corn (8.7% CP)	63.6	54.2
Soybean meal (44.2% CP)	20.5	21.1
Wheat bran (14.3% CP)	4.80	10.6
Soybean oil	0.00	3.00
Limestone	8.50	8.50
Calcium hydrogen phosphate	1.04	1.04
Sodium chloride	0.38	0.38
DL-Methionine	0.18	0.18
Vitamin-mineral premix^1^	1.00	1.00
Total	100	100
**Calculated nutrient composition (%)**		
Metabolic energy (MJ/kg)	11.4	11.4
Crude protein (CP)	15.3	15.6
Calcium	3.37	3.37
Total phosphorus	0.53	0.55
Available phosphorus	0.30	0.31
Lysine	0.72	0.73
Methionine	0.41	0.41

1 *Vitamin–mineral premix provided the following per kilogram of mixed feed: Mn, 50.18 mg; Zn, 40.22 mg; Fe, 40.14 mg; Cu, 6.10 mg; I, 0.29 mg; Se, 0.18 mg; vitamin A, 12,500 IU; vitamin D3, 3,750 IU; vitamin E, 15 IU; vitamin K3, 2.5 mg; vitamin B1, 2.5 mg; vitamin B2, 7.0 mg; vitamin B6, 3.75 mg; vitamin B12, 0.015 mg; folic acid, 1.0 mg; pantothenic acid, 12.5 mg; niacin, 25 mg; and biotin, 0.075 mg*.

### Determination of Growth Performance and Feed Intake

Five laying hens in each replicated cage (*n* = 30 per group) were randomly selected to measure body weight at 25, 29, 33, and 37 WOA, and average body weight of birds in each group was calculated based on the individual weight. For the determination of feed intake of laying hens in two groups, total feed intake of birds in per replicated cage was recorded daily for 6 consecutive days before bone sample collection at 25, 29, 33, and 37 WOA, respectively. Average daily feed intake was calculated according to the ratio of total feed intake to the number of birds.

### Determination of Production Performance and Egg Quality

Production performance likes the number of eggs and broken eggs were recorded for 5 consecutive days at 25, 29, 33, and 37 WOA, respectively. The average rates of egg production and broken egg were separately calculated based on the data collected each day. For the determination of egg quality, two eggs from each cage (*n* = 12 per group) were randomly collected to measure parameters, including egg weight, eggshell strength and thickness, albumen height and Haugh unit. Eggshell strength (kg/cm^3^) was determined using an egg force reader (ORKA-ESTG-1; ORKA Technology Ltd, Ramat Asharon, Israel). Haugh unit and albumen height were determined using an egg-quality gauge (EMT-5200, Japan).

### Assessment of Keel Bone Damage

Five laying hens in each cage (*n* = 30 per group) were randomly selected for assessment of keel bone status by palpation at 25, 29, 33, and 37 WOA, following a method described previously (12). For the purpose of this study, keel bone status was classified into three categories: normal, deviated, or fractured. If a bird presented both keel bone deviation and fracture, fractured keel bone status was assigned, as fracture is more likely to induce pain than deviation. The percentage of KBD in laying hens in each tested group at each sampling point was determined based on aforementioned diagnostic palpation.

### Keel Bone Sample Collection

After assessment of KBD at each time-point, 12 laying hens in each group (*n* = 2 each cage) were randomly selected and slaughtered by cervical dislocation. Correspondingly, their keel bones were excised from the body, muscle and other soft tissues connected to the keel bone were removed, and keel bone length was determined with a digital caliper. All excised keel bones were subsequently stored in −80°C freezers until further analysis.

### Micro Computed Tomography (Micro-CT) Analysis

Dissected keel bones from laying hens of 29 and 37 WOA in both evaluated groups (*n* = 6 from each group per sampling point) were sliced to 1.3 cm pieces in length, and subsequently soaked in 4% paraformaldehyde solution for Micro-CT analysis. Keel bone samples were imaged in a micro-CT scanner (μCT 50; Scanco Medical AG., Zürich, Switzerland) at 16 μm voxel size, 70 kV source potential, 200 μA current, 300 ms integration time, and 0.5 mm aluminum filter. Each scan was restructured using Mimics 19.0. Advanced bone analysis (ABA) software to determine the following parameters of bone microstructure: BV/TV, Tb.Th, Tb.Sp, Tb.N, bone surface (BS), bone surface/bone volume (BS/BV), DA, Conn.Dn, and BMD.

### Determination of Major and Trace Elements in Keel Bone

Keel bone samples from laying hens of 33 WOA (*n* = 6 per tested group) were defrosted at 4°C and ground. Subsequently, 1.0 g of sample was treated overnight with 5 mL of 65% (w/w) HNO_3_, 2 mL of 30% H_2_O_2_ (w/v), and 3 mL of deionized water. Samples were submitted to microwave digestion at 100°C for 3 min, 150°C for 10 min, and 180°C for 45 min. After cooling to room temperature, digested samples were brought to 50 mL final volume with Milli-Q water and mixed well prior to each determination. Concentration of 29 the following major and trace elements was determined: lithium (Li), B, sodium (Na), magnesium (Mg), aluminum (Al), silicon (Si), P, potassium (K), Ca, titanium (Ti), vanadium (V), chromium (Cr), manganese (Mn), iron (Fe), cobalt (Co), nickel (Ni), Cu, zinc (Zn), arsenic (As), selenium (Se), strontium (Sr), molybdenum (Mo), cadmium (Cd), stannum (Sn), antimony (Sb), barium (Ba), mercury (Hg), thallium (Tl), and lead (Pb). Determination of bone elements was performed using inductively coupled plasma mass spectrometry (ICP-MS) (iCAP™ Q ICP-MS; Thermo Fisher Scientific, Waltham, MA, USA) according to manufacturer's instructions. Optimized parameters used in ICP-MS analysis are presented in Table 2. Concentration of bone mineral elements was determined based on blank and known standards.

**Table 2:** Parameter conditions for inductively coupled plasma mass spectrometry (ICP-MS).

**Parameter (unit)**	**Condition**
Frequency (Hz)	27.1
Reflect power (W)	1550
Carrier gas flow rate (L/min)	1.05
Plasma gas flow rate (L/min)	14.0
Auxiliary gas rate (L/min)	0.80
Spray chamber temperature (°?)	2.70
Sampling depth (mm)	6.00
Nebulizer pump (r/min) Oxide ions (156/140, %) Doubly charged (70/140, %)	40.0 <2.0 <3.0

### RNA Isolation and Gene Quantification by Real-Time PCR (qRT-PCR)

Total RNA was obtained from keel bone excised from laying hens at each sampling point (*n* = 6 per group and sampling point) using RN54-EASY-spin Plus Bone Tissue RNA Kit according to manufacturer's instructions (Aidlab Biotechnologies Ltd., Beijing, China). Obtained RNA was eluted in 40 μL of 0.1% RNase free water, and RNA concentration and purity were determined at 260 nm and 260/280 nm using a GeneQuant 1300/GeneQuant 100 spectrophotometer (Harvard Bioscience, Inc., Holliston, MA, USA). cDNA was synthesized from 2 μg of total RNA using oligo (dT) primers, Superscript II reverse transcriptase and PrimeScript™ RT Reagent Kit with gDNA Eraser (TaKaRa Bio USA, Inc., Dalian, China). Synthesized cDNAs were five-fold diluted in sterile water and stored at −80°C for further qRT-PCR analysis.

PCR oligonucleotide sequences of all surveyed genes are listed in Table 3. qRT-PCR analysis was conducted in a LightCycler^®^ 480 Real-Time PCR System (Roche, Basel, Switzerland). Each 10 μL reaction mixture contained 0.3 μL of each primer (10 mM), 1 μL of diluted cDNA, 5 μL of 2× Roche Fast Universal SYBR Green Master kit (Roche), and 3.4 μL of PCR-grade water. qRT-PCR conditions were as follows: pre-denaturation at 95°C for 2 min, followed by 40 cycles of denaturation at 95°C for 15 s, annealing and extension at 60°C for 1 min. For each gene, qRT-PCR reactions were performed in triplicates, and threshold cycle (Ct) value was calculated based on a mean of triplicate values. The 2^−ΔΔCt^ method was used to calculate the relative mRNA expression level of each gene, with housekeeping gene β-actin as internal reference.

**Table 3:** Primer sequences of each gene for qRT-PCR.

**Gene**	**GenBank ID**	**Primers sequence (5′ → 3′)**
OPG	NM_001033641.1	Forward: ATCTCAGTCAAGTGGAGCATC Reverse: GTTCCAGTCTTCAGCGTAGTA
RANKL	NM_001083361.1	Forward: AGGAGAAATAAGCCCGAGAA Reverse: TTTGTTATGATGCCAGGATGTA
COL2α1	NM_001079714	Forward: GGCTTTGATGCAGAATACTACCG Reverse: GTTGTTCAATGTTTTCAGAGTGGC
SOST	XM_004948551.2	Forward: CGTCCTCATCCAAATCGCATCCC Reverse: GCCTGGTTCATCGTGTTGTTGTTG
POSTN	NM_001030541	Forward: TGGAGCTGTCTATGAAACTTTGG Reverse: GAGTGTATTGGCCTTCTGGTCTT
β-actin	NM_205518.1	Forward: CACGATCATGTTTGAGACCTT Reverse: CATCACAATACCAGTGGTACG

*OPG, osteoprotegerin; RANKL, receptor activator of nuclear factor kappa-B ligand; COL2α1, collagen type II alpha 1; POSTN, periostin; SOST, sclerostin*.

### Statistical Analysis

Statistical analysis was performed in SPSS 22 software (SPSS Inc., Chicago, IL, United States). Normality of data was determined using the Kolmogorov-Smirnov test, and independent *t*-test was then used to compare differences in bone length, bone structural parameters, mineral elements concentration, and expression of genes involved in bone quality in normal and fractured bone samples at each time-point. Production performance (egg production and broken egg rate), egg quality (egg weight, egg thickness and breaking strength, albumen height, and Haugh unit), body weight, and daily feed intake data were analyzed by a repeated measures analysis, and each replicated cage was considered as an experimental unit. Owing to the data of production performance, daily feed intake, body weight, and egg quality meet the Mauchly's Test of Sphericity, and there is no interaction between the test time-points and groups, one-way ANOVA was applied to compare their difference with Duncan's multiple comparison. Results were expressed as mean and SEM, and differences were considered statistically significant when *P* ≤ 0.05.

## Results

### Determination of Body Weight, Production Performance and Feed Intake

As shown in Table 4, egg production and broken egg rate in laying hens were not evidently different (*P* > 0.05) between both CON group and SO group at each time-point. Body weight of laying hens in SO group was higher (*P* < 0.05) than CON group at 29 and 37 WOA, but no significant difference was found in both groups at 25 and 33 WOA (*P* > 0.05). Average daily feed intake of laying hens in SO group at 33 and 37 WOA was lower (*P* < 0.05) than that in CON group, but no significant difference was found in both groups at 25 and 29 WOA (*P* > 0.05).

**Table 4:** Body weight, daily feed intake and production performance of laying hens fed a commercial diet (control group, CON) or a soybean oil-supplemented diet (SO).

**Items**	**Trail weeks**	**CON**	**SO**	***P*-value**
**Body weight (kg)**				
	25	1.22 ± 0.03	1.26 ± 0.02	0.24
	29	1.38 ± 0.03	1.47 ± 0.03	0.04
	33	1.50 ± 0.04	1.54 ± 0.04	0.34
	37	1.54 ± 0.03	1.65 ± 0.02	0.01
**Daily feed intake (g)**				
	25	126 ± 1.41	126 ± 1.22	0.98
	29	129 ± 0.96	127 ± 1.72	0.29
	33	131 ± 0.79	125 ± 1.04	<0.01
	37	131 ± 0.96	126 ± 1.39	0.01
**Egg production (%)**				
	25	42.4 ± 1.22	42.0 ± 1.21	0.83
	29	70.2 ± 1.42	71.6 ± 0.75	0.43
	33	82.3 ± 1.48	84.6 ± 1.34	0.28
	37	85.2 ± 1.21	85.8 ± 1.70	0.78
**Broken egg rate (%)**				
	25	6.50 ± 0.90	6.61 ± 0.58	0.93
	29	2.52 ± 0.60	4.36 ± 0.59	0.06
	33	0.91 ± 0.61	2.72 ± 0.55	0.06
	37	0.71 ± 0.43	1.39 ± 0.64	0.41

*Values were expressed as the mean ± SEM*.

### Determination of Egg Quality

Egg quality parameters in laying hens from CON and SO groups are shown in Table 5. Compared to CON group, egg weight, eggshell breaking strength, eggshell thickness, albumen height, and Haugh unit were not significantly different in SO group at each test time-point (*P* > 0.05).

**Table 5:** Comparison of egg quality in laying hens fed a commercial diet (control group, CON) or a soybean oil-supplemented diet (SO).

**Items**	**Trail weeks**	**CON**	**SO**	***P*-value**
**Egg weight (g)**				
	25	51.8 ± 1.10	51.8 ± 0.79	0.97
	29	52.3 ± 1.37	52.6 ± 0.92	0.87
	33	55.4 ± 1.09	56.7 ± 1.07	0.39
	37	60.8 ± 1.18	63.9 ± 1.32	0.08
**Eggshell strength (kg/cm**^**3**^**)**				
	25	4.03 ± 0.07	4.13 ± 0.11	0.44
	29	4.16 ± 0.09	4.33 ± 0.12	0.27
	33	4.24 ± 0.13	4.44 ± 0.11	0.25
	37	4.61 ± 0.16	4.67 ± 0.12	0.76
**Eggshell thickness (mm)**				
	25	0.39 ± 0.01	0.39 ± 0.01	0.76
	29	0.37 ± 0.01	0.39 ± 0.01	0.29
	33	0.38 ± 0.01	0.38 ± 0.01	0.91
	37	0.40 ± 0.01	0.39 ± 0.01	0.47
**Albumen height (mm)**				
	25	5.83 ± 0.31	5.85 ± 0.25	0.97
	29	6.54 ± 0.24	7.02 ± 0.29	0.21
	33	7.70 ± 0.12	7.88 ± 0.29	0.58
	37	8.31 ± 0.22	8.56 ± 0.26	0.49
**Haugh unit**				
	25	63.6 ± 3.85	65.5 ± 2.83	0.69
	29	69.9 ± 4.04	72.3 ± 3.20	0.65
	33	82.5 ± 2.60	84.5 ± 2.63	0.59
	37	89.5 ± 1.37	91.5 ± 1.05	0.27

*Values were expressed as the mean ± SEM, n = 12*.

### Assessment of Keel Bone Damage

**Figure 1:**
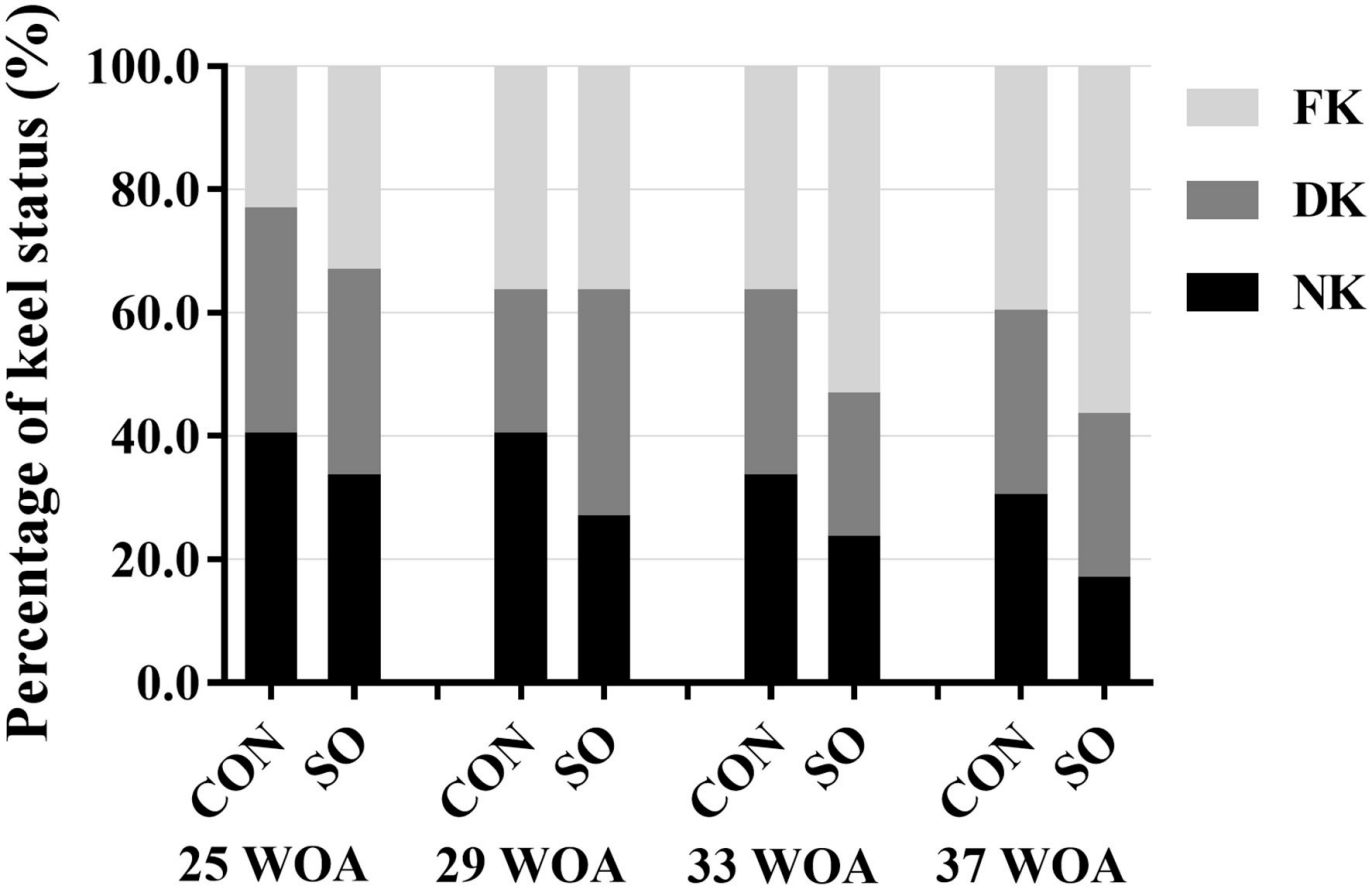
Percentages of laying hens fed a commercial diet (control group, CON) or a soybean oil-supplemented diet (SO) presenting normal keel bone (NK), deviated keel bone (DK), and fractured keel bone (FK) at different sampling points.

The results of KBD evaluation in laying hens in CON and SO groups are shown in Figure 1. The frequency of normal keel bone in laying hens in SO group was lower than in CON group at all sampling points. In contrast, the incidence of fractured keel bone in SO group was higher than CON group at 25, 33, and 37 WOA; however, incidences were similar at 29 WOA. The occurrence of deviated keel bone in SO group was lower than CON group at 25, 33, and 37 WOA, but increased at 29 WOA. Overall, compared to CON group, the occurrence of normal keel bone in birds fed a SO-supplemented diet was decreased while that of fractured keel bone was increased.

### Determination of Keel Bone Length

Variations in keel bone length in laying hens from CON and SO groups at each sampling point are shown in Table 6. Compared to CON group, keel bone length in laying hens of SO group was significantly reduced (*P* < 0.05) at all sampling points.

**Table 6:** Length (mm) of keel bone of laying hens in control (CON) and soybean oil (SO) groups.

	**CON**	**SO**	***P*-value**
25 weeks of age	100 ± 0.89	96.8 ± 1.04	0.03
29 weeks of age	100 ± 1.12	96.8 ± 0.87	0.03
33 weeks of age	102 ± 0.84	97.1 ± 1.86	0.03
37 weeks of age	103 ± 1.00	97.8 ± 1.17	0.01

*Values were expressed as mean ± SEM, n = 12*.

### Determination of Keel Bone Microstructure and Mineral Density

Values of keel bone microstructure parameters and BMD in laying hens of both tested groups at 29 and 37 WOA are presented in Table 7. Keel Tb.N and BMD were significantly reduced (*P* < 0.05) while Tb.Sp was increased (*P* < 0.01) in hens of SO group compared to CON group at 29 WOA. Tb.N and Tb.Th were significantly decreased (*P* < 0.05), and BMD was significantly decreased (*P* < 0.01) in hens of SO group at 37 WOA.

**Table 7:** Keel bone microstructure parameters and bone mineral density of laying hens in control (CON) and soybean oil (SO) groups.

	**29 weeks of age**			**37 weeks of age**		
**Item (unit)**	**CON**	**SO**	***P*-value**	**CON**	**SO**	***P*-value**
BV/TV (%)	0.73 ± 0.05	0.70 ± 0.05	0.68	0.66 ± 0.04	0.70 ± 0.07	0.65
BS/BV (1/mm)	6.73 ± 0.63	7.11 ± 0.48	0.64	6.73 ± 0.60	6.32 ± 1.21	0.77
Tb.N (1/mm)	5.14 ± 0.16	4.36 ± 0.24	0.03	4.41 ± 0.32	3.51 ± 0.25	0.05
Tb.Th (mm)	0.36 ± 0.10	0.23 ± 0.01	0.28	0.27 ± 0.02	0.21 ± 0.02	0.04
Tb.Sp (mm)	0.16 ± 0.01	0.20 ± 0.01	0.01	0.21 ± 0.02	0.27 ± 0.03	0.12
Conn.Dn (1/mm^3^)	29.2 ± 4.03	21.9 ± 3.19	0.19	22.6 ± 2.30	16.8 ± 1.65	0.07
DA	1.91 ± 0.23	1.60 ± 0.06	0.22	1.96 ± 0.14	1.75 ± 0.10	0.25
BMD (mg HA/cm^3^)	643 ± 12.1	611 ± 5.14	0.04	633 ± 12.5	585 ± 8.48	0.01

*Values were expressed as mean ± SEM, n = 6*.

*BV/TV, bone volume/tissue volume; BS/BV, bone surface/bone volume; Tb.N, trabecular number; Tb.Th, trabecular thickness; Tb.Sp, trabecular separation; Conn.Dn, connectivity density; DA, degree of anisotropy; BMD, bone mineral density*.

### Determination of Mineral Elements Content in Keel Bone

Contents of mineral elements in keel bone obtained from laying hens in both tested groups at 33 WOA are shown in Table 8. Compared to CON group, contents of Li, Ca, Hg, and Tl in SO group were significantly reduced (*P* < 0.05); in contrast, contents of V, Mn, Fe, Se, and Ba were increased (*P* < 0.05). Contents of other surveyed elements were not significant different between tested groups (*P* > 0.05).

**Table 8:** Elements concentration in keel bones of laying hens in control (CON) and soybean oil (SO) groups.

**Element**	**Unit**	**CON**	**SO**	***P*-value**
Li	mg/kg	0.17 ± 0.01	0.11 ± 0.01	0.01
B	mg/kg	1.07 ± 0.06	1.06 ± 0.03	0.83
Na	g/kg	5.56 ± 0.41	5.42 ± 0.28	0.79
Mg	g/kg	2.50 ± 0.21	2.48 ± 0.22	0.95
Al	mg/kg	9.77 ± 1.34	7.33 ± 0.55	0.13
Si	mg/kg	235 ± 9.66	225 ± 12.3	0.51
P	g/kg	121 ± 12.5	120 ± 10.7	0.93
K	g/kg	3.29 ± 0.07	4.18 ± 0.49	0.11
Ca	g/kg	368 ± 10.9	323 ± 14.2	0.04
Ti	g/kg	0.64 ± 0.05	0.81 ± 0.06	0.07
V	mg/kg	0.30 ± 0.02	0.40 ± 0.04	0.04
Cr	mg/kg	3.96 ± 0.56	4.72 ± 0.58	0.37
Mn	mg/kg	7.08 ± 0.55	9.38 ± 0.28	0.01
Fe	mg/kg	96.7 ± 7.18	130 ± 8.62	0.02
Co	μg/kg	25.6 ± 2.32	22.7 ± 0.69	0.26
Ni	mg/kg	0.34 ± 0.02	0.30 ± 0.03	0.31
Cu	mg/kg	0.69 ± 0.03	0.75 ± 0.06	0.46
Zn	mg/kg	143 ± 4.44	155 ± 11.9	0.35
As	μg/kg	50.3 ± 3.39	45.6 ± 2.37	0.28
Se	mg/kg	0.23 ± 0.02	0.28 ± 0.01	0.05
Sr	mg/kg	93.6 ± 2.58	96.1 ± 2.26	0.47
Mo	mg/kg	0.19 ± 0.01	0.20 ± 0.01	0.05
Cd	μg/kg	3.00 ± 0.62	2.16 ± 0.27	0.25
Sn	mg/kg	0.39 ± 0.01	0.39 ± 0.01	0.99
Sb	mg/kg	0.19 ± 0.01	0.20 ± 0.01	0.36
Ba	mg/kg	21.0 ± 1.35	27.0 ± 1.68	0.02
Hg	μg/kg	1.21 ± 0.11	0.83 ± 0.12	0.04
Tl	μg/kg	1.49 ± 0.05	0.85 ± 0.07	<0.01
Pb	mg/kg	0.36 ± 0.06	0.65 ± 0.16	0.13

*Values were expressed as the mean ± SEM, n = 6*.

### Expression of Genes Related to Keel Bone Quality

**Figure 2:**
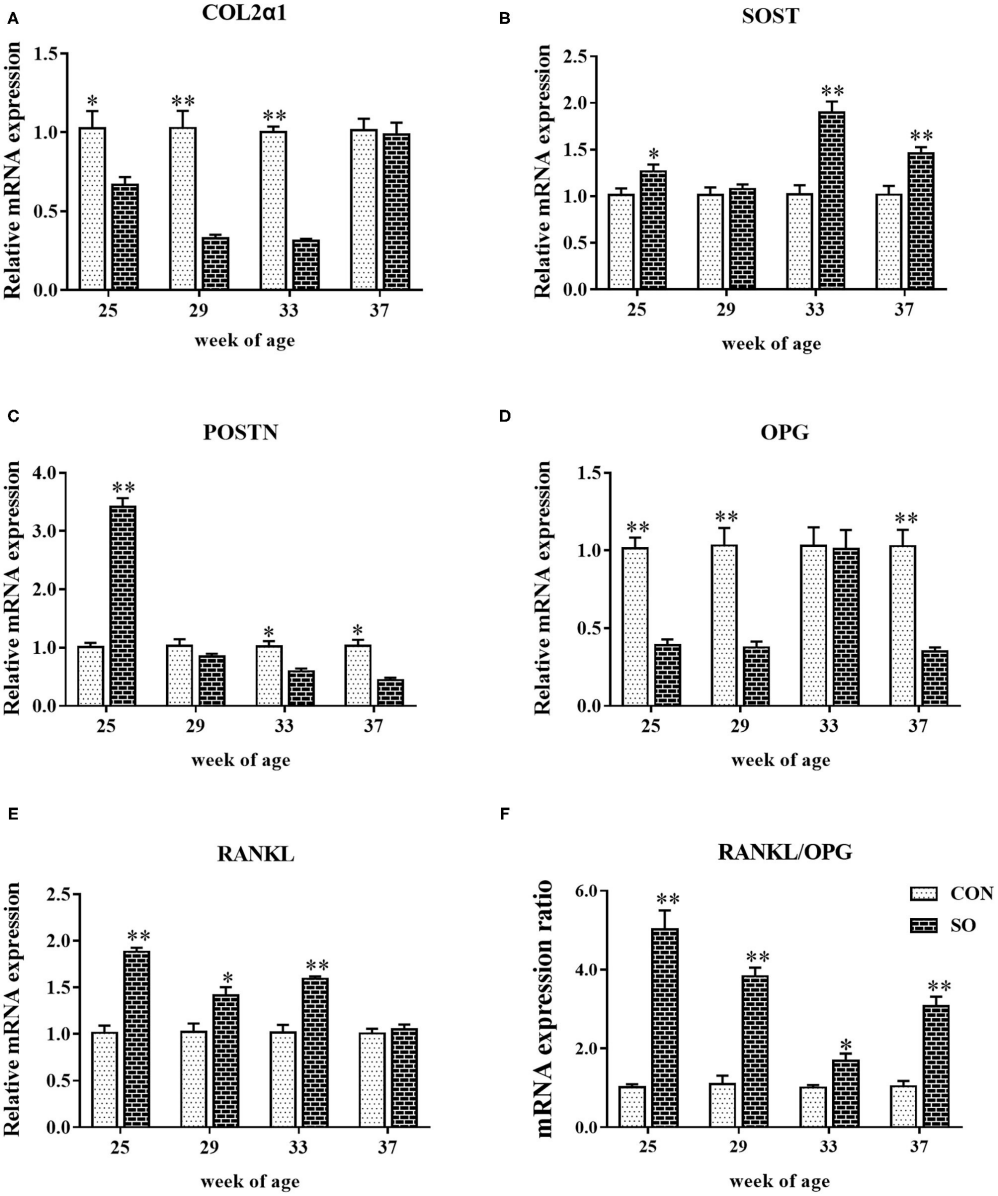
Relative mRNA levels of **(A)** COL1α2, **(B)** SOST, **(C)** POSTN, **(D)** OPG, **(E)** RANKL, and **(F)** ratio of RANKL/OPG mRNA expression in keel bones of laying hens fed a commercial diet (control group; CON) and a soybean oil-supplemented diet (SO). Values are expressed as mean ± SEM, *n* = 6. *P < 0.05, **P < 0.01.

Relative mRNA levels of COL2α1, OPG, RANKL, SOST, and POSTN in keel bones from hens in CON and SO groups are shown in Figure 2. At 25, 29, and 33 WOA, mRNA levels of COL2α1 were significantly decreased (*P* < 0.05), while expression of RANKL was significantly decreased (*P* < 0.05) in SO group. mRNA levels of SOST in SO group were increased compared to CON group at 25, 33 and 37 WOA (*P* < 0.05); mRNA expression of OPG was downregulated in SO group at 25, 29 and 37 WOA (*P* < 0.01). mRNA expression of POSTN in SO group at 25 WOA was significantly upregulated (*P* < 0.01), whereas at 33 and 37 WOA it was significantly downregulated (*P* < 0.05) compared to CON group. Ratio of RANKL/OPG mRNA expression in SO group was higher than that in CON group at all sampling points (*P* < 0.05).

## Discussion

Oil-enriched diets are often applied to poultry industry due to it can provide more energy and facilitate the absorption of nutrients. Previous studies reported that dietary SO supplementation (3.5% or more) increased egg weight and daily egg production in laying hens (2). Furthermore, Küçükersan et al. found that egg weight and egg production were also improved by fed hens with 3% SO-diet (3). In the present study, elevated body weight was found in laying hens fed a 3% SO-diet, but egg quality parameters and production performance were not different between CON group and SO group. In addition, our study showed that laying hens fed 3% SO-diet could decrease average daily feed intake in the later stages of the experiment. This finding is similar to the results of Jiang et al., who studied on laying hens found that the diet with over 7% SO, daily feed intake was significantly reduced (7). Thus, this study indicated that SO-enriched diet decreased daily feed intake and increased body weight in laying hens.

KBD is prevalent in laying hens in all production systems. Layer strain and age, as well as nutrition levels affect the incidence of KBD (20, 21). Previous studies with laying hens fed a diet high in SO content (over 10%) revealed increased bone loss and alterations in tibial strength and mineralization from early-stage laying (7). In the present study, dietary supplementation with 3% of SO increased the occurrence of KBD (especially fracture) in laying hens throughout the evaluated period, thus impairing on keel bone health. We speculated that increased the incidence of KBD in laying hens fed SO-diet that could be related to decreased daily feed intake, because reduced feed intake will lead to a low level of Ca intake, thus weakening bone mineralization and strength, and elevating bone damage. Furthermore, keel bones in laying hens fed a SO-supplemented diet were shorter in length, which suggests that supplementation with soybean oil may impair keel bones development during the laying period.

Parameters of bone microstructure as revealed by Micro-CT imaging are strongly correlated with bone health status (22). BV/TV, Tb.Th, Tb.Sp, Conn.Dn, and DA have been described to play an obvious role in bone resistance to fracture. High values of BV/TV, Tb.Th, Tb.N, and low values of Conn.Dn and BS/BV are correlated with high levels of bone mechanical strain (23). Tb.Th is also positively correlated with bone strength. Additionally, measurement of BMD has become a suitable predictor of fracture risk as it reflects bone strength (14). Recent studies have shown that dietary Mn deficiency reduces tibia bone quality by decreasing Tb.N, Tb.Th, and increasing Tb.Sp in broilers (24). Dietary cadmium chloride supplementation impairs bone metabolism by increasing Tb.Sp and decreasing Tb.N in laying hens (25). Probiotic addition into the diet of laying hens improved tibia and femur quality by increasing BMD and bone mineral content (26). In the present study, supplementation with 3% of SO to the diet given to laying hens reduced keel bone quality as revealed by decreased levels of Tb.N, Tb.Th, BMD, and increased levels of Tb.Sp. These findings are corroborated by the results of Jiang et al., who described femoral and tibial loss as a result of dietary SO supplementation (7), thus reinforcing its link with impaired bone quality in laying hens. Furthermore, determination of BMD can precisely indicate changes in gain and loss of bone mass, serving as a bone mineralization indicator in animals (14). The results presented herein showed that keel bone mineral density in laying hens in SO group was significantly reduced compared to CON group, which suggests bone mass loss or decreased bone mineralization due to a SO-enriched diet. In line with these observations, da Costa et al. reported that adult male rats fed a SO-supplemented diet had reduced bone strength and mass as indicated by decreased femur and lumbar BMD (27). Collectively, data indicate that a SO-enriched diet can have detrimental effects on keel bone mineralization and quality in laying hens.

Minerals are ubiquitous inorganic nutrients in tissues and fluids, and whose presence is necessary for the maintenance of certain physicochemical processes in human, animals and plants (28). Minerals can be classified as macro (including Ca, P, Na, and chloride), micro (such as Fe, Cu, Co, K, Mg, Zn, Mn, Mo, Cd, Se, etc.) and ultra-micro (including B, Si, As, and Ni) elements based on corresponding required amounts (28). Many studies reported that dietary Ca supplementation could improve bone quality by increasing bone homeostasis, BMD, and bone strength in laying hens and broilers (6, 29). In this study, however, dietary SO supplementation decreased keel bone concentration of Ca and BMD in laying hens, thus indicating that SO can have a negative effect on bone calcium deposits and quality. Mn is the most abundant micro-element found in bone, accounting for 43% of total body Mn (30). However, excessive amounts of Mn can lead to toxic effects on many organs in humans and animals at different stages of development (30). In the present study, Mn content in keel bone of laying hens fed a SO-rich diet was significantly higher compared to CON group, despite bone quality being lower. This finding may suggest that the significant increase in Mn content induced by SO supplementation may be toxic to laying hens and impair keel bone health. Fe is an essential element for bone health and development as it participates in a wide variety of metabolic processes. Fe overload or deficiency is positively correlated with bone loss due to an imbalanced bone resorption and formation (31), which in turn indicates that a well-balanced bone homeostasis requires optimal Fe contents. In the present study, the increased levels of Fe in keel bone of hens receiving a SO-enriched diet are more likely to promote bone resorption, which may lead to bone loss and reduced bone mass.

Se is a micro-element involved in mitigating the effects of oxidative stress and improving bone health by reducing bone resorption. Previous studies indicated that Se supplementation reduced the Cd-induced risk of KBD in laying hens (32). From above discussed results, it can be hypothesized that an increased Se content as a result of SO supplementation may have played a protective role against KBD induced by high levels of Mn and Fe in laying hens. Se may have counterbalanced Fe-stimulated bone resorption, thus reducing bone damage. In this study, laying hens fed a SO-supplemented diet showed higher levels of V, which may influence bone homeostasis. In a study using rats as a model, it has been shown that V accumulates in the bones, thus affecting bone metabolism by direct stimulation of osteoblasts (33). Previous studies also showed that higher Li levels inhibited bone resorption and affected BMD, and Li supplementation decreased risk of fractures by influencing bone anabolic properties (34). In the present study, decreased levels of Li in keel bones of layering hens had an adverse effect on bone health and quality by altering its metabolism and mineral density. Additionally, Tl and Hg are toxic elements to animals and humans (35). Low levels of Tl and Hg were identified in keel bone of layering hens in SO group, which indicates that SO supplementation can alleviate Tl and Hg toxic effects on bones. Therefore, changes in the levels of Li, Ca, Hg, Tl, V, Mn, Fe, Se, and Ba as induced by a SO-enriched diet affected bone turnover and mineral homeostasis in laying hens.

RANKL and OPG are produced by osteoblast-lineage cells, which are considered to play an important role connecting bone formation and resorption during bone remodeling (36). RANKL promotes differentiation and activation of osteoclasts, as well as maintains and stimulates bone resorption activity, while OPG suppresses bone resorption and remodeling and increases bone mass (17). Studies in meat ducks revealed that low-nutrient-density diets with 0.7% or more of Ca content led to an increased expression of OPG, but had no influence on RANKL expression, thus indicating that it contributes to the maintenance of optimal bone mass by suppressing bone resorption (37). The results discussed herein showed that RANKL mRNA levels in keel bone of laying hens fed a SO-supplemented diet were increased, whereas expression of OPG was reduced, which indicates that dietary SO supplementation may accelerate bone resorption and induce bone loss. The ratio of RANKL/OPG mRNA levels also plays a dominant role in osteoclastogenesis, as it regulates osteoclast formation, and a high ratio is suggestive of bone loss (36). Prior studies in broilers linked Mn deficiency with a significant high ratio of RANKL/OPG mRNA levels in tibia, which further induced accelerated differentiation of osteoclasts, increased osteoclast activity and bone loss (24). Similarly, a high ratio of RANKL/OPG mRNA levels was found in keel bones of laying hens fed with a SO-supplemented diet, indicating that SO enrichment may induce keel bone loss. COL2α1 controls bone structural framework and strength, being associated with bone remodeling at transcriptional level. Spent laying hens with perch access were shown to have altered femur bone remodeling as demonstrated by upregulated mRNA expression of COL2α1 (18). In the present study, mRNA and protein levels of COL2α1 in keel bone of laying hens fed a SO-rich diet were decreased, which indicates impairment of keel bone framework and strength. In addition, parameters used to evaluate keel bone microstructure and associated with bone strength and stiffness, such as Tb.N, Tb.Th, Tb.Sp, and BMD, were altered to various degrees in laying hens in SO group. Thus, reduced keel bone quality is likely to be related to downregulation of COL2α1.

SOST and POSTN are single nucleotide polymorphisms known to be involved in controlling bone health and quality. Prior studies in laying hens demonstrated that mRNA level of SOST can be a good predictive biomarker for bone quality variation, such as in osteoporosis (38), and SOST can decrease bone formation by reducing osteoblastic activity. In the present study, the expression of SOST was increased in keel bone of hens in SO group, which suggests that dietary supplementation with SO likely induced alterations in keel bone quality in a process involving SOST. POSTN is an extracellular matrix protein involved in bone quality by regulating osteoblast differentiation and bone formation. Laying hens fed diets containing insufficient amounts of Ca were shown to possess higher levels of POSTN (19), thus indicating that an increase in POSTN mRNA expression is probably associated with a restorative activity on most damaged bone microstructures. Additionally, Zhang et al. reported that the expression of POSTN is essential for bone formation induced by mechanical forces and fractures in mice (39). However, in the present study, expression of POSTN was increased in keel bone of laying hens in SO group at 25 WOA and decreased at subsequent sampling points, suggesting that high POSTN expression may be related to the repair of damaged keel bone microstructure at early adoption of SO supplementation. Moreover, later decreased POSTN expression could be associated with imbalanced bone formation and absorption caused by long-term feeding with SO. da Costa et al. reported that diets containing SO had a negative impact on bone health and strength in rats (27). In the present study, long-term feeding laying hens with SO-supplemented diet led to impaired keel bone health and quality, which might be linked with decreased POSTN expression, since it also controls bone metabolism and quality by regulating osteoblast differentiation and bone formation.

Additionally, in the present study, dietary SO supplementation induced mainly keel bone damage that may be related to the changes in fatty acid (e.g., omega-3 and omega-6) composition in the diet. Previous reports showed that dietary added omega-3 fatty acid decreased the incidence of keel bone breakage and improved bone health and quality by elevated bone breaking strength, bone mineral density and bone turnover in laying hens (21). Besides, omega-3 and omega-6 ratio is also important to maintain optimal bone health in poultry (40). Thus, further research should consider the effects of omega-3 content and omega-3 and omega-6 ratio in the diet on keel bone quality in laying hens.

## Conclusions

In conclusion, the findings discussed herein indicated that dietary supplementation with 3% of SO mainly increased the incidence of keel bone damage, impaired keel bone development, decreased average daily feed intake and bone mineral density, and altered bone microstructure and mineral elements homeostasis, as well as expression of genes related to bone quality in laying hens housed in enriched cages. Therefore, feeding hens a diet enriched with 3% of SO can lead to negative effects on feed intake and keel bone health and quality but not affects egg production and egg quality during the laying period.

## Data Availability Statement

The original contributions presented in the study are included in the article/supplementary material, further inquiries can be directed to the corresponding author/s.

## Ethics Statement

All experimental protocols and the use of animals were approved by and conducted according to the guidelines of the Institutional Animal Care and Use Committee of Northeast Agriculture University (NEAU-[2011]-9).

## Author Contributions

JB conceived and supervised the study. HW and JB designed the experiments. HW, LP, CL, and PZ performed the experiments. HW wrote the manuscript. JL, RZ, and JB revised and edited the manuscript. All authors read and approved the final manuscript.

## Conflict of Interest

The authors declare that the research was conducted in the absence of any commercial or financial relationships that could be construed as a potential conflict of interest.
